# A comparison of risk factors for metastasis at diagnosis in humans and dogs with osteosarcoma

**DOI:** 10.1002/cam4.2177

**Published:** 2019-04-21

**Authors:** Brandon J. Diessner, Tracy A. Marko, Ruth M. Scott, Andrea L. Eckert, Kathleen M. Stuebner, Ann E. Hohenhaus, Kim A. Selting, David A. Largaespada, Jaime F. Modiano, Logan G. Spector

**Affiliations:** ^1^ Division of Pediatric Epidemiology and Clinical Research School of Medicine University of Minnesota Minneapolis Minnesota; ^2^ Department of Pediatrics School of Medicine University of Minnesota Minneapolis Minnesota; ^3^ Masonic Cancer Center University of Minnesota Minneapolis Minnesota; ^4^ Department of Veterinary Clinical Sciences College of Veterinary Medicine University of Minnesota St Paul Minnesota; ^5^ Animal Cancer Care and Research Program University of Minnesota St Paul Minnesota; ^6^ Animal Medical Center Bobst Hospital New York New York; ^7^ Veterinary Medical Teaching Hospital University of Missouri Columbia Missouri; ^8^ Center for Immunology University of Minnesota Minneapolis Minnesota; ^9^ Stem Cell Institute University of Minnesota Minneapolis Minnesota; ^10^ Institute for Engineering in Medicine University of Minnesota Minneapolis Minnesota; ^11^ Present address: Regions Hospital St Paul Minnesota; ^12^ Present address: Riversbend Animal Hospital Big Rapids Michigan; ^13^ Present address: Department of Veterinary Clinical Medicine University of Chicago Urbana Illinois

**Keywords:** dog, human, metastasis, osteosarcoma

## Abstract

**Background:**

Canine osteosarcoma (OS) is a relevant spontaneous model for human OS. Identifying similarities in clinical characteristics associated with metastasis at diagnosis in both species may substantiate research aimed at using canine OS as a model for identifying mechanisms driving distant spread in the human disease.

**Methods:**

This retrospective study included dog OS cases from three academic veterinary hospitals and human OS cases from the Surveillance, Epidemiology, and End Results program. Associations between clinical factors and metastasis at diagnosis were estimated using logistic regression models.

**Results:**

In humans, those with trunk tumors had higher odds of metastasis at diagnosis compared to those with lower limb tumors (OR = 2.38, 95% CI: 1.51, 3.69). A similar observation was seen in dogs with trunk tumors compared to dogs with forelimb tumors (OR = 3.28, 95% CI 1.36, 7.50). Other associations were observed in humans but not in dogs. Humans aged 20‐29 years had lower odds of metastasis at diagnosis compared to those aged 10‐14 years (OR = 0.67, 95% CI: 0.47, 0.96); every 1‐cm increase in tumor size was associated with a 6% increase in the odds of metastasis at diagnosis (95% CI: 1.04, 1.08); compared to those with a white, non‐Hispanic race, higher odds were observed among those with a black, non‐Hispanic race (OR: 1.51, 95% CI: 1.04, 2.16), and those with a Hispanic origin (OR 1.35, 95% CI: 1.00, 1.81).

**Conclusion:**

A common mechanism may be driving trunk tumors to progress to detectable metastasis prior to diagnosis in both species.

## 1 INTRODUCTION

Osteosarcoma (OS) is the most commonly diagnosed primary bone malignancy in children, adolescents, and young adults.^1^ An estimated 15% to 20% of the patients present with detectable metastasis at diagnosis,^2^, ^3^ which portends a significantly poorer prognosis compared to presenting with localized disease.^2^, ^4^, ^5^, ^6^, ^7^ Despite treatment of primary tumor resection and high‐dose chemotherapy, only 20% to 40% of patients with detectable metastasis at diagnosis will be long‐term disease‐free survivors.^3^, ^7^, ^8^

With a peak annual incidence of eight cases per million in the adolescent and young adult population, research aimed at identifying the underlying mechanisms driving metastatic progression is challenged by obtaining a sufficient number of patients to study.^9^ In contrast, spontaneous OS is much more common in dogs, with an estimated annual incidence of at least 139 cases per million dogs,^10^ and the similarities between canine OS and human OS, including natural occurrence, clinical presentation, prognostic factors, genetic aberrations, dysregulation of key molecular pathways, biological behaviors, and metastatic progression,^11^, ^12^, ^13^, ^14^, ^15^, ^16^, ^17^, ^18^, ^19^, ^20^, ^21^, ^22^ suggest that conserved pathways are required to develop the OS phenotype.^12^ However, OS primarily occurs in mid‐aged to older dogs after growth plates have closed,^23^ a distinct difference between the human and canine disease.

Despite the dissonant ages at peak incidence, identifying similarities in patient and tumor characteristics associated with metastasis at diagnosis in both species may substantiate further research aimed at using canine OS as a model for identifying mechanisms necessary for distant spread in the human disease. The purpose of this study was to identify and compare clinical factors associated with the presence of metastasis at diagnosis in humans and dogs with OS.

## 2 MATERIALS AND METHODS

### 2.1 Eligible cases and data extraction from human database

All cases under 30 years of age presenting with high grade OS were identified in the Surveillance, Epidemiology, and End Results (SEER) program database using “OS ICD‐O‐3 codes” (9180, 9181, 9182, 9183, 9185, 9186, 9187, 9192, 9193, 9194) (n = 1960). Only cases presenting with their first primary malignancy between 2004 and 2015 were considered for analysis (n = 1862). Metastatic OS was characterized based on a SEER staging variable (“DerivedSS2000”). A staging of “distant” was classified as presenting with distant metastasis (n = 402), while cases with a staging of “localized” or “regional” were not (n = 1391). Cases for which staging was unknown were excluded from analysis (n = 69).

Patient characteristics of interest included age, sex, primary tumor site, primary tumor size, and race/ethnicity. Age was categorized into groups that were defined a prior (<10 years, 10‐14 years, 15‐19 years, and 20‐29 years). Cases were categorized as having a Hispanic origin based on a SEER variable,^24^ whereas those without evidence of a Hispanic origin were subcategorized into mutually exclusive groups based on their reported race. Those reported as white or black were categorized as “White, Non‐Hispanic” or “Black, Non‐Hispanic,” respectively, and those with a reported race of either “Asian or Pacific Islander” or “American Indian/Alaskan Native” were grouped together as “API or AI/AN.” Primary tumor sites were grouped by anatomical location based on ICD‐O‐3 site codes: lower limb (C402‐3), upper limb (C400‐1), trunk (C412‐4), and head (C410‐1). Primary sites with nonspecific classifications or those from areas outside of bone (eg, heart or liver) were classified as missing (n = 19). Cases with either exact tumor size measurements (n = 1531) or nonspecific size descriptions (eg, <2 cm; n = 6) were categorized into three broad groupings (<5 cm, 5‐10 cm, >10 cm), as has been done elsewhere.^25^ Primary tumor size measurements were missing for 256 cases.

### 2.2 Eligible cases and data extraction from canine databases

A retrospective review of electronic medical records was conducted to identify pet dogs diagnosed with OS from the University of Minnesota Veterinary Medical Center (n = 364), Animal Medical Center in New York (n = 113), and the University of Missouri (n = 324), representing the largest known cohort of dogs analyzed for associations between clinical variables and metastasis at diagnosis (Table 1). Dogs with either a histopathologically confirmed diagnosis or a presumptive diagnosis of OS from imaging and/or cytology between 2003 and 2017 were eligible for inclusion into this study. Dogs with evidence of gross metastasis at diagnosis from imaging, biopsy, or necropsy results were categorized as presenting with distant metastasis (n = 85), while those without such evidence were not (n = 600). Dogs for which evidence of metastasis at diagnosis was unknown were excluded from further analysis (n = 116).

**Table 1:** Univariate associations of dog and tumor characteristics across veterinary hospitals

	Veterinary clinic [N (%)]			*P*‐value	N
	AMC	MU	UMN		
Chronological age (y)				<0.001	685
<6	5 (5)	51 (17)	35 (12)		
6‐10	32 (34)	166 (54)	165 (58)		
>10	57 (61)	91 (30)	83 (29)		
Physiological age (y)^a^				<0.001	685
<40	4 (4)	38 (12)	20 (7)		
40‐60	31 (33)	165 (54)	170 (60)		
>60	14 (15)	105 (34)	85 (30)		
Missing	45 (48)	0 (0)	8 (3)		
Sex				0.316	685
Female	47 (50)	164 (53)	133 (47)		
Male	47 (50)	144 (47)	150 (53)		
Tumor location				<0.001	685
Forelimb	36 (38)	177 (58)	149 (53)		
Hindlimb	25 (27)	89 (29)	109 (39)		
Trunk	14 (15)	18 (6)	11 (4)		
Head	19 (20)	24 (8)	12 (4)		
Missing	0 (0)	0 (0)	2 (1)		
Tumor size (cm)				<0.001	685
<5	20 (21)	10 (3)	7 (2)		
5‐10	20 (21)	13 (4)	44 (16)		
>10	6 (6)	6 (2)	34 (12)		
Missing	48 (51)	279 (91)	198 (70)		
Body weight (kg)				<0.001	685
<22	42 (45)	29 (9)	16 (6)		
22‐45	7 (7)	186 (60)	193 (68)		
>45	0 (0)	93 (30)	66 (23)		
Missing	45 (48)	0 (0)	8 (3)		
Breed				<0.001	685
Golden	9 (10)	30 (10)	50 (18)		
Labrador	11 (12)	47 (15)	69 (24)		
Rottweiler	8 (9)	32 (10)	27 (10)		
Other	66 (70)	199 (65)	137 (48)		
Metastasis at diagnosis				0.656	685
No	84 (89)	266 (86)	250 (88)		
Yes	10 (11)	42 (14)	33 (12)		

Abbreviations: AMC, Animal Medical Center; MU, University of Missouri; UMN, University of Minnesota.

*Note* Some proportions do not add to 100 because of rounding.

a Dog age in human year equivalents.

Characteristics of interest were similar to humans, including age, sex, primary tumor location, primary tumor size, as well as body weight and breed. Body weight was grouped into three body weight categories (small: <22 kg, medium: 22‐45 kg, and large: >45 kg), and was missing for 53 dogs. Chronological dog age was categorized into three groups (<6 years, 6‐10 years, and >10 years). However, because breed and body weight can influence the longevity of pet dogs, we standardized the chronological age of dogs in terms of equivalent physiological age in human years.^26^ Once transformed, dog age was also categorized into three broad physiological age categories in human year equivalents (<40 years, 40‐60 years, and >60 years). Seventy‐six breeds were available for analysis. The three most common were categorized as reported (Labrador retrievers, golden retrievers, or Rottweilers), and the 73 other breeds were grouped together as “other.” Tumor location groupings were based on the reported primary tumor location and were made to parallel the human categories. Primary tumor location was unknown for two dogs. Thirty‐two (5%) sexually intact males, 15 (2%) sexually intact females, and four (1%) dogs for which neutered status was unknown were grouped with neutered or spayed dogs as either “Male” or “Female,” respectively. For tumor size, the largest dimension of absolute tumor length, absolute tumor width, or absolute tumor height was categorized into one of the three tumor size categories used for human tumor size measurements (<5 cm, 5‐10 cm, >10 cm). Accurate tumor measurements were available for 160 dogs with known staging at diagnosis. Cut‐off values for body weight and age are were arbitrarily defined a priori.

### 2.3 Statistical methods

Chi square and Fisher's exact tests were used to evaluate univariate associations in humans and dogs. Complete‐case logistic regression models were created to identify clinical factors independently associated with metastatic OS at presentation in each species. Unless otherwise noted, odds ratios for humans were adjusted for age at diagnosis, sex, primary tumor site, primary tumor size, and race/ethnicity; odds ratios for dogs were adjusted for physiological age in human year equivalents, sex, primary tumor site, body weight, and breed. We did not adjust logistic regression models in dogs by veterinary hospital because a similar proportion of dogs presented with metastasis at diagnosis across hospitals, and including it as a covariate in the fully adjusted logistic regression model did not alter associations more than 10% (data not shown). Additionally, tumor size was not included in the canine logistic regression model because tumor size measurements were only available for 23% of the dogs with known tumor staging. Whenever possible, reference levels for the canine logistic regression models were set to parallel those set for humans. For tumor location categories, we hypothesized that the same mechanical and functional stresses on weight‐bearing bones that are thought to contribute to OS primary tumor risk may also influence metastatic development.^27^ As such, the reference level for primary tumor location was set to “lower limbs” in humans and “forelimbs” in dogs since the forelimbs bear approximately 60% of a dog's weight.^28^

Missing data in both the human and dog datasets were handled by multiple imputation assuming that the unobserved data were missing at random (Table S1). Five imputed datasets were created for each species. The variables used for the imputation models were the same as those used for the analytic models, except that “veterinary hospital” was added to the imputation model for dogs. Results from logistic regression models with and without imputation produced similar parameter estimates and 95% CI. Therefore, results are presented only from the complete case analysis.

We also performed an ad hoc analysis on a subset of 223 dogs from the University of Missouri with data on duration of clinical signs before diagnosis. Using a Mann‐Whitney *U* test, we evaluated the difference in the median time to diagnosis between dogs with trunk tumors and dogs with limb tumors (hind limb and forelimb tumors combined).

All statistics were calculated using R version 3.2.4.^29^ All alpha levels presented are two‐sided.

## 3 Results

### 3.1 Metastasis by primary tumor location

Metastasis at diagnosis was observed in 22% of the humans and 12% of the dogs with OS. In both species, primary tumors located in the trunk had the highest prevalence of metastasis (36% in humans and 23% in dogs), whereas primary tumors located in the head of humans (9%) and the forelimbs of dogs (10%) had the lowest. In the univariate analysis (Table 2), primary tumor location was significantly associated with metastasis at diagnosis in humans (*P* < 0.001); the *P*‐value for the association in dogs was 0.10. In the multivariate analyses (Table 3), primary tumors located in the trunk in both species had the highest odds of presenting with metastasis. The odds of metastasis at diagnosis from the other primary tumor location categories were not significantly different from each species respective references.

**Table 2:** Univariate analysis of key clinical factors and metastatic osteosarcoma at diagnosis in humans and dogs

Humans				Dogs			
Characteristic	Total	Metastatic disease at diagnosis	*P*‐value	Characteristic	Total	Metastatic disease at diagnosis	*P*‐value
Age (y)			0.13	Chronological age (y)			0.86
<10	230	47 (20)		<6	91	11 (12)	
10‐14	581	148 (25)		6‐10	363	43 (12)	
15‐19	566	126 (22)		>10	231	31 (13)	
20‐29	416	81 (19)		Physiological age (y)^a^			0.48
Sex			0.15	<40	62	9 (15)	
Female	812	169 (21)		40‐60	366	43 (12)	
Male	981	233 (24)		>60	204	31 (15)	
Tumor site			<0.001	Sex			0.15
Head	86	8 (9)		Female	344	36 (10)	
Lower limb	1314	281 (21)		Male	341	49 (14)	
Trunk	136	49 (36)		Tumor site			0.1
Upper limb	238	57 (24)		Head	55	6 (11)	
Tumor size (cm)			<0.001	Forelimb	362	38 (10)	
<5	185	16 (9)		Trunk	43	10 (23)	
5‐10	648	115 (18)		Hind limb	223	30 (13)	
>10	704	196 (28)		Tumor size (cm)			0.52
Race/ethnicity			0.02	<5	37	5 (13)	
White, NH	823	160 (19)		5‐10	77	8 (10)	
Black, NH	281	69 (25)		>10	46	8 (17)	
API or AI/AN	110	31 (22)		Body weight (kg)			0.26
Hispanic	399	142 (26)		<22	87	13 (15)	
				22‐45	386	44 (11)	
				>45	159	26 (16)	
				Breed			0.27
				Golden	89	9 (10)	
				Labrador	127	17 (13)	
				Rottweiler	67	13 (19)	
				Other	402	46 (11)	
				Veterinary hospital			0.66
				AMC	94	10 (11)	
				MU	308	42 (14)	
				UMN	283	33 (12)	

Abbreviations: AI/AN American Indian/Alaskan Native; AMC, Animal Medical Center; API, Asian/Pacific Islander; MU, University of Missouri; NH, Non‐Hispanic; UMN, University of Minnesota.

a Dog age in human year equivalents.

**Table 3:** Adjusted odds ratio and 95% CI from logistic regression analysis for metastatic osteosarcoma (OS) at diagnosis in humans and dogs

Humans (n = 1506)		Dogs (n = 630)	
Characteristic	OR (95% CI)	Characteristic	OR (95% CI)
Age (y)		Physiological age (y)^a^	
<10	0.9 (0.59, 1.35)	<40	1.48 (0.63, 3.15)
10‐14	Ref	40‐60	Ref
15‐19	0.82 (0.6, 1.13)	>60	1.48 (0.88, 2.46)
20‐29	0.67 (0.47, 0.96)		
Sex		Sex	
Female	Ref	Female	Ref
Male	1.29 (0.99, 1.67)	Male	1.51 (0.93, 2.46)
Tumor site		Tumor site	
Head	0.54 (0.2, 1.21)	Head	1.27 (0.43, 3.27)
Lower limb	Ref	Forelimb^b^	Ref
Trunk	2.38 (1.51, 3.69)	Trunk	3.28 (1.36, 7.5)
Upper Limb	1.21 (0.83, 1.73)	Hind limb	1.49 (0.87, 2.53)
Tumor size		Body weight (kg)	
1‐cm increase	1.06 (1.04, 1.08)	<22	1.27 (0.58, 2.65)
Race/ethnicity		22‐45	Ref
White, NH	Ref	>45	1.59 (0.89, 2.8)
Black, NH	1.51 (1.04, 2.16)	Breed	
API or AI/AN	1.1 (0.66, 1.77)	Golden	0.71 (0.27, 1.71)
Hispanic	1.35 (1.00, 1.81)	Labrador	Ref
		Rottweiler	1.52 (0.64, 3.52)
		Other	0.84 (0.45, 1.64)

Abbreviations: AI/AN, American Indian/Alaskan Native; API, Asian/Pacific Islander; NH, Non‐Hispanic.

a Dog age in human year equivalents.

b Reference levels for tumor site were set to lower limb in humans and forelimb in dogs to account for similarities in weight‐bearing and mechanical forces that are hypothesized to contribute to OS risk.

### 3.2 Metastasis by sex

A similar proportion of males and females appeared to present with metastasis at diagnosis in both species (univariate *P*‐values: Human = 0.15; Dog = 0.15), and the 95% CI for the ORs in both species contained the null in the multivariate analyses (Humans OR: 1.29, 95% CI: 0.99, 1.67; Dogs OR: 1.51, 95% CI: 0.93, 2.46).

### 3.3 Metastasis by age at diagnosis

In the multivariate analysis, humans diagnosed with OS between the ages of 20 to 29 had significantly lower odds of presenting with metastasis compared to those diagnosed between the ages of 10‐14 years (OR: 0.67 95% CI: 0.47, 0.96). Physiological age in human year equivalents was not significantly associated with metastasis in the multivariate analysis of dogs.

### 3.4 Metastasis by human ancestry

Those with a black, non‐Hispanic race, or a Hispanic origin had the highest observed prevalence of metastasis at diagnosis (25% and 26%, respectively, *P* = 0.02). Compared to those with a white, non‐Hispanic race, the odds of metastasis was 35% higher in those with a Hispanic origin (95% CI: 1.00, 1.81) and 51% higher in those with a black, non‐Hispanic race (95% CI: 1.04, 2.16).

### 3.5 Metastasis by dog body weight and breed

Neither dog body weight nor breed was associated with metastasis at diagnosis.

### 3.6 Metastasis by primary tumor size

Tumor size was significantly associated with metastasis at diagnosis in humans (*P* < 0.001), but not dogs with available tumor size measurements (*P* = 0.52). When added to the human logistic regression analysis as a continuous variable, we observed a 6% increase in the odds of metastatic disease at diagnosis for every 1‐cm increase in tumor size (95% CI: 1.04, 1.08).

### 3.7 Duration of signs before OS diagnosis in dogs

In the ad hoc analysis, we observed that dogs that presented with trunk tumors (n = 17) had a shorter median duration of signs before a diagnosis compared to dogs that presented with tumors in the limbs (n = 206) (median time: 2 weeks vs 4 weeks, respectively. *P* = 0.03; Data not shown).

## 4 DISCUSSION

Our analysis found that older age (20‐29 years vs 10‐14 years), trunk tumors, larger tumor size, and having a reported black, non‐Hispanic race, or Hispanic ethnicity were independently associated with metastatic OS at diagnosis in human cases under 30 years of age, and that trunk tumors were independently associated with metastatic OS at diagnosis in dogs.

Other studies have found that primary tumor size^25^, ^30^, ^31^ and primary tumor location^25^, ^32^, ^33^, ^34^ were associated with metastatic OS at diagnosis in humans. Miller and colleagues found similar associations between clinical factors and metastasis at diagnosis in human cases from the SEER database, but their analysis focused on patients of all ages with an emphasis on older adults (>60 years).^25^ Our analysis builds upon their work by identifying risk factors in patients <30 years of age, who represent the majority of OS cases (~60% in SEER database). Additionally, by evaluating risk factors for metastasis at diagnosis in dogs, we aimed to further substantiate using OS in dogs as a spontaneous model for early‐onset OS in humans. Our findings suggest a common mechanism may be driving a subset of OS tumors to progress to detectable metastases prior to initial diagnosis in both species, despite the dissonant ages at peak incidence.

OS primarily affects adult dogs after growth plate closure, a distinct difference noted between the canine and human disease^23^ and further validated here (dog median age: 8.8 years, interquartile range 7‐10.4 years). Nevertheless, risk of OS is still speculated to be associated in part with skeletal development and growth in dogs, as it is in humans. Long periods of tumor latency can precede clinical detection, as is observed in lung cancer of former smokers,^35^ such that growth plate closure before tumor development does not preclude a role for skeletal remodeling and growth in the pathogenesis of OS in dogs. Indeed, it is widely reported that canine OS most commonly occurs in the appendicular skeleton of large and giant dogs, affecting the forelimbs, which bear more of a dog's body weight, nearly twice as often as the hind limbs.^14^, ^20^, ^36^, ^37^ Our data support these observations, with 91% of the tumors in dogs >22 kg occurring in the appendicular skeleton, arising 1.68‐times more frequently in the forelimbs compared to the hind limbs. However, risk of OS cannot entirely be ascribed to body size parameters, since variable rates of OS incidence among breeds of similar size have been reported.^36^ Notably, we observed a wide range of body weights among the most common breeds in our analysis (min kg, median kg, max kg: golden retriever: 15, 36, 58; Labrador retriever: 13, 37, 57; Rottweiler: 15, 44, 63; Data not shown), further suggesting that heritable traits contribute to risk of OS in dogs independent of size. Similar observations are noted in humans with OS, who are generally in higher percentiles of size for their age,^38^ but may also be genetically predisposed from inherited cancer syndromes.^39^ It is also the case that the proportion of OS tumors arising in the weight‐bearing bones of the appendicular skeleton in children, adolescents, and young adults is similar to that observed in dogs.^14^

With regard to OS metastasis at diagnosis, our results also indicate that similar mechanisms may be influencing risk in both species. We did not observe a difference in the odds of metastasis at diagnosis between the appendicular tumor location categories in either species, implying that the mechanical and functional stresses on weight‐bearing bones that are hypothesized to contribute to OS primary tumor risk might not substantially influence metastatic development. This is further evidenced by the observation we and others have found that the odds of metastasis were significantly greater among tumors arising in the bones of trunk,^25^, ^32^, ^40^, ^41^, ^42^, ^43^ which are under substantially less stress from motion and weight‐bearing compared to the long appendicular bones.^44^

Instead, our observations with tumor location as well as age at diagnosis suggest that skeletal tissue vascularization may be influential in early‐onset of gross OS metastasis in dogs and humans. In young people and dogs, a majority of bone marrow is comprised of highly vascularized hematopoietic red bone marrow.^45^, ^46^ Though variations exist, red marrow converts to relatively hypovascular yellow marrow beginning in the immediate postnatal period until approximately 25 years in humans and 3 years in dogs, when red marrow is predominantly concentrated in the axial skeleton.^45^, ^46^ OS tumors that develop either in childhood or in the bones of the trunk may therefore be more likely to arise near highly vascularized skeletal tissue, making it easier for malignant cells to intravasate into blood vessels. This may also explain why we observed an association with trunk tumors, but not age at diagnosis in dogs, since the majority of OS tumors in dogs develop after attainment of adult bone marrow patterns.

Successful metastasis also requires intravasated cells to survive circulation, extravasate, and colonize distant microenvironments.^47^ Whether the molecular traits required to do so are intrinsic to a subset of OS tumors or sequentially acquired from continuous tumor proliferation and adaption to the selective pressures of distant spread remains unclear. Trunk OS tumors cause few early signs and symptoms in both dogs and humans,^32^, ^40^, ^48^, ^49^ implying that these tumors are perhaps afforded an extended period of time prior to diagnosis to acquire the molecular changes needed to successfully disseminate malignant cells. However, we observed that dogs with trunk tumors actually had a shorter duration of signs before a diagnosis compared to dogs with appendicular tumors, and the association with trunk tumors observed in human OS cases was independent of tumor size, which may serve as a proxy for time to diagnosis and tumor proliferation. Moreover, if metastatic traits are sequentially acquired over time, our results imply that OS patients diagnosed in childhood or early adolescence face a longer time to diagnosis compared to young adults. Yet studies have shown the opposite to be true,^50^, ^51^ perhaps because young adults are under reduced parental surveillance or have limited knowledge of their own physical health.^52^, ^53^ Instead, certain OS tumors in both humans and dogs may be biologically predetermined to successfully metastasize, regardless of time to diagnosis.^18^, ^54^ Using both human and dog OS, future research should ascertain whether evolutionarily conserved pathways are needed to develop the metastatic OS phenotype, and whether patterns of gene expression could be used to categorize intrinsically aggressive molecular subtypes of OS in both species.

In humans, we also observed increased odds of metastasis among cases with a Hispanic origin or a black, non‐Hispanic race. Similar to our other observations, it is unclear whether such results arose from extrinsic factors that could delay diagnosis. In the United States, race/ethnicity is highly correlated with socioeconomic status (SES),^55^ and previous analyses of SEER data has shown that counties with the lowest composite SES scores have a higher proportion of patients with metastasis at diagnosis.^25^ It is also the case that the prevalence of metastasis at diagnosis is higher in countries with lower human development index scores, where there are more barriers to accessing appropriate healthcare.^56^ Conversely, a recent mediation analysis of several pediatric cancers reported that SES could not fully account for the survival disparities across different racial groups, indicating that other factors, including intrinsic tumor biology, may also play a role.^57^ To our knowledge, intrinsic differences in the biological character of OS tumors between populations have not yet been reported, but a single nucleotide polymorphism in the NFIB gene was recently associated with a significantly increased odds of presenting with metastasis.^58^ Notably, the frequency of the risk allele (*A*) is higher in a population with Mexican ancestry in Los Angeles (22%) compared to populations of European ancestry (14%).^59^ Although any risk ascribed to different racial/ethnic groups is likely interplay of both extrinsic and biological factors, fully understanding the observed associations requires further research aimed at understanding whether there exists any differences in intrinsic biological character of OS tumors across populations.

There are several limitations with this study. First, the limited availability of tumor size measurements for dogs precluded us from including it in the multivariate model. Additionally, we did not standardize available tumor size measurements to the relative size of the dog and could not distinguish between breed size and body condition (relative proportion of fat, muscle, and bone) in our analysis of dog body weight. The self‐reporting of dog breeds may have resulted in breed misclassification, though previous research has shown the accuracy of breed self‐reports to be >87% and could be >95%.^60^ Also, our analysis of time to diagnosis in dogs relied on the owner's first recognition of signs. It could therefore be the case that owner's simply recognized signs of OS in dogs with trunk tumors later in the stage of their disease compared to dogs with appendicular tumors. It is also the case that the accuracy of methods used to detect metastasis differed between humans and dogs. In humans, metastasis at diagnosis was based on a SEER summary stage variable derived from the collaborative stage algorithm that utilizes the most precise clinical and pathological information obtained from medical records.^61^ Conversely, metastasis in dogs was mostly assessed from radiography, which has been shown to be inferior to other techniques.^62^, ^63^, ^64^ Thus, a subset of metastatic OS tumors in dogs may have been misclassified as nonmetastatic. We speculate that this misclassification occurred independent of other prognostic variables and therefore may have biased our results toward the null.^65^ The accuracy of tumor size measurements in dogs is also a limitation of our analysis, since measurements are known to vary depending upon the imaging modality used.^66^, ^67^ Furthermore, the absence of a confirmed OS diagnosis from histopathology results is a limitation inherent in our dog data. We therefore include dogs with a presumptive diagnosis of OS from imaging or cytology. Cytology has been associated with high accuracy, yet imaging remains insufficient as a single inclusion criterion.^68^ Thus, it is possible that a subset of dogs included in our analysis were misdiagnosed with OS. In the human analysis, there may also have been misclassification of race/ethnicity. However, SEER race/ethnicity classifications have been reported to have excellent agreement with self‐reported racial classifications, except for AI/AN.^69^

Understanding why the prevalence of metastasis at diagnosis differs by important clinical factors may have significant implications for patient care and risk stratification, and the similarities observed in both species suggests that a central mechanism may be defining the differences observed. Utilizing the metastatic OS canine model, which resembles human metastatic OS, has greater incidence, and undergoes more rapid progression, may help identify new treatment strategies to improve survival in both humans and pet dogs.

## CONFLICT OF INTEREST

The authors declare no potential conflict of interest.

## AUTHOR CONTRIBUTIONS

BJD involved in conceptualization, formal analysis, data curation, validation, writing—original draft, visualization, and project administration; TAM involved in conceptualization, validation, writing—original draft, and project administration; RMS, ALE, and KMS involved in investigation, writing—review, and editing; AEH and KAS involved in investigation, resources, validation, writing—original draft, and visualization; DAL involved in conceptualization, resources, writing—review and editing, and supervision; JFM and LGS involved in conceptualization, resources, validation, writing—original draft, visualization, and supervision.
