# Supplementary material for: A comparison of risk factors for metastasis at diagnosis in humans and dogs with osteosarcoma
Source: Cancer Med. 2019 Apr 21;8(6):3216–26. doi: 10.1002/cam4.2177 (PMC6558582; doi:10.1002/cam4.2177)
Supplement: Supplementary file 1 [file CAM4-8-3216-s001.docx]

| **Supplementary Table 1. Adjusted odds ratio and 95% CI from logistic regression analysis for metastatic osteosarcoma at diagnosis in humans and dogs using imputed datasets** | | | | |
| --- | --- | --- | --- | --- |
| **Humans (N = 1,862)** | |  | **Dogs (N = 801)** | |
| **Characteristic** | OR (95% CI) |  | **Characteristic** | OR (95% CI) |
| **Age (yrs)** |  |  | **Physiological age (yrs)†** | |
| < 10 | 0.83 (0.57, 1.22) |  | < 40 | 0.72 (0.33, 1.57) |
| 10 - 14 | REF |  | 40 - 60 | REF |
| 15 - 19 | 0.78 (0.58, 1.04) |  | > 60 | 0.95 (0.42, 2.11) |
| 20 - 29 | 0.68 (0.49, 0.94) |  |  |  |
| **Sex** |  |  | **Sex** |  |
| Female | REF |  | Female | REF |
| Male | 1.19 (0.93, 1.52) |  | Male | 1.41 (0.88, 2.26) |
| **Tumor site** |  |  | **Tumor site** |  |
| Head | 0.62 (0.29, 1.33) |  | Head | 1.26 (0.48, 3.26) |
| Lower limb | REF |  | Forelimb‡ | REF |
| Trunk | 2.45 (1.66, 3.61) |  | Trunk | 2.65 (1.13, 6.22) |
| Upper limb | 1.28 (0.92, 1.79) |  | Hind limb | 1.38 (0.82, 2.32) |
| **Tumor size** |  |  | **Body weight (kg)** |  |
| 1 cm increase | 1.06 (1.04, 1.08) |  | < 22 | 1.12 (0.45, 2.79) |
| **Race/Ethnicity*** |  |  | 22 - 45 | REF |
| White, NH | REF |  | > 45 | 1.65 (0.66, 4.13) |
| Black, NH | 1.35 (0.96, 1.88) |  | **Breed** |  |
| API or AI/AN | 1.19 (0.76, 1.88) |  | Golden | 1.33 (0.54, 3.26) |
| Hispanic | 1.42 (1.09, 1.84) |  | Labrador | REF |
|  |  |  | Rottweiler | 2.16 (0.73, 6.35) |
|  |  |  | Other | 1.19 (0.56, 2.56) |
| Abbreviations: CI, confidence intervals; Yrs,Years; NH, Non-Hispanic; API, Asian/Pacific Islander; AI/AN, American Indian/ Alaskan Native; cm, centimeter; kg, kilogram. † Dog age in human year equivalents; ‡Reference levels for tumor site were set to lower limb in humans and forelimb in dogs to account for similarities in weight bearing and mechanical forces that are hypothesized to contribute to OS risk. | | | | |
